# Thrombotic microangiopathy following systemic AAV administration is dependent on anti-capsid antibodies

**DOI:** 10.1172/JCI173510

**Published:** 2024-01-02

**Authors:** Stephanie M. Salabarria, Manuela Corti, Kirsten E. Coleman, Megan B. Wichman, Julie A. Berthy, Precilla D’Souza, Cynthia J. Tifft, Roland W. Herzog, Melissa E. Elder, Lawrence R. Shoemaker, Carmen Leon-Astudillo, Fatemeh Tavakkoli, David H. Kirn, Jonathan D. Schwartz, Barry J. Byrne

**Affiliations:** 1 Department of Pediatrics, University of Florida, Gainesville, Florida, USA.; 2 National Human Genome Research Institute, NIH, Bethesda, Maryland, USA.; 3 Indiana University, South Bend, Indiana, USA.; 4 4D Molecular Therapeutics, Emeryville, California, USA.; 5 Rocket Pharma, Cranbury, New Jersey, USA.

**Keywords:** Genetics, Immunology, Innate immunity

## Abstract

**BACKGROUND:**

Systemic administration of adeno-associated virus (AAV) can trigger life-threatening inflammatory responses, including thrombotic microangiopathy (TMA), acute kidney injury due to atypical hemolytic uremic syndrome–like complement activation, immune-mediated myocardial inflammation, and hepatic toxicity.

**METHODS:**

We describe the kinetics of immune activation following systemic AAV serotype 9 (AAV9) administration in 38 individuals following 2 distinct prophylactic immunomodulation regimens. Group 1 received corticosteroids and Group 2 received rituximab plus sirolimus in addition to steroids to prevent anti-AAV antibody formation.

**RESULTS:**

Group 1 participants had a rapid increase in immunoglobulin M (IgM) and IgG. Increase in D-dimer, decline in platelet count, and complement activation are indicative of TMA. All Group 1 participants demonstrated activation of both classical and alternative complement pathways, as indicated by depleted C4 and elevated soluble C5b-9, Ba, and Bb antigens. Group 2 patients did not have a significant change in IgM or IgG and had minimal complement activation.

**CONCLUSIONS:**

This study demonstrates that TMA in the setting of AAV gene therapy is antibody dependent (classical pathway) and amplified by the alternative complement pathway. Critical time points and interventions are identified to allow for management of immune-mediated events that impact the safety and efficacy of systemic gene therapy.

## Abstract 2

Thrombotic microangiopathy in the setting of adeno-associated virus gene therapy is antibody dependent as part of the classical complement pathway.

## Graphical abstract

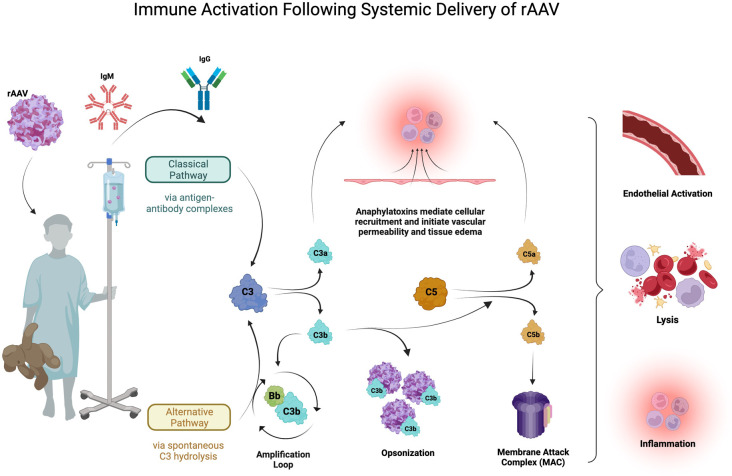

## Introduction

Adeno-associated virus–mediated (AAV-mediated) gene therapy has emerged as a promising treatment approach for a spectrum of monogenic genetic disorders (1–4). With the approval of the first recombinant AAV vector–based systemic gene therapy, onasemnogene abeparvovec (Zolgensma) for the treatment of spinal muscular atrophy (SMA), the versatility and clinical success of this approach are evident (3). However, while these therapies offer a new approach to treat devastating diseases, the immunologic responses to AAV vectors pose a unique challenge that affects their safety and efficacy (5–7). The activation of the innate and adaptive immunologic responses after de novo exposure to AAV, as well as humoral and cellular responses to preexisting host immunity, are some of the primary barriers to the expanded use of these therapies (8–10).

AAV particles are a potent activator of the innate immune system, not only through the physical properties of the capsid particle, but also through the response to the high capsid protein load associated with systemic AAV administration (11–13). Pathogen-associated molecular patterns in AAV are recognized via the adaptor proteins, Toll-like receptors (TLRs), primarily TLR2 and TLR9, which can trigger an innate immune response and promote the activation of adaptive immunity through activation of cytokines and interferons (IFNs) that in turn activate CD8^+^ T cells (8, 12, 14, 15). It is important to note that cytokine production is influenced by the Fc γ receptors (FcγRs), which recognize the Fc region of immunoglobulin G (IgG). FcγRIIa is the main cytokine-inducing receptor in humans, which influences activation of TLR9, resulting in the production of IFN-α as well as other cytokines and chemokines such as TNF-α, IL-1β, IL-6, and IL-8 (16). AAV exposure can trigger the complement system, an innate defensive mechanism that induces rapid destruction of pathogens and acts as a functional sensor of the surface area of invading particles (17–19). The complement system is composed of more than 30 proteins that play important roles in recognition and elimination of pathogens (20). Complement activation by AAV is primarily antibody dependent (classical pathway), triggered by anti-capsid IgM and IgG antibodies that can cause complement-mediated cell damage (18). However, studies using human samples both in vivo and in vitro demonstrate that complement can be activated by direct interaction of C3 protein and AAV capsid proteins (alternative pathway) (8, 11, 21). Zaiss et al. demonstrated that AAV capsid particles interact with the complement proteins C3, C3b, iC3b, and complement regulatory factor H (11, 12, 22, 23). All pathways result in the formation of the C3 convertases (C4b2b), which cleave C3 into C3a and C3b. C3b binds to C4b2b and creates C5 convertase (C4b2b3b). C5 convertase produces the most potent small peptide mediator of inflammation, C5a, and the large active fragment, C5b, which initiates the late events of complement activation. C5b binds to C6, C7, C8, and C9 to generate the soluble C5b-9 (SC5b-9) membrane attack complex (MAC), leading to cell lysis and cell death (17, 24). Both C5a and MAC can cause acute hepatic and myocardial injury (25). Liver injury is reflected in the early elevation of aspartate aminotransferase (AST) and alanine aminotransferase (ALT) following systemic AAV exposure. Similarly, vector-antibody complexes are deposited on endothelia and cardiomyocytes. The MAC’s mechanism of cell membrane perforation is similar across affected cells, and may cause contemporaneous troponin leakage and AST/ALT elevation.

Evidence from ongoing clinical trials (ClinicalTrials.gov NCT03368742, NCT04281485, and NCT03882437) suggests that high doses (5 × 10^13^ to 2 × 10^14^ vg/kg) of AAV significantly increase complement activation. Some participants in these studies presented with severe and life-threatening inflammatory responses that were likely secondary to the activation of the complement system (12, 26–30). In addition to nausea, fever, and vomiting likely due to cytokine release, participants presented with complement-mediated thrombotic microangiopathy (CM-TMA) (31), acute kidney injury due to atypical hemolytic uremic syndrome–like (aHUS-like) complement activation, thrombocytopenia, and immune-mediated myocardial injury (28, 31–33). Activation of complement is a major safety consideration for gene therapy, as growing evidence suggests that high-dose intravenous (i.v.) AAV infusion or high exposure to AAV empty capsids leads to antibody-dependent activation of the complement system in human plasma (34). Additionally, local tissue activation of complement should be considered a consequence of improved AAV capsid specificity based on capsid development and evolution, which may deposit higher levels of AAV in targeted tissue beds.

Cases of life-threatening complement activation have been managed with hemodialysis, platelet transfusion, and attempts to blunt the complement-mediated adverse events with C5 inhibition via eculizumab, a monoclonal antibody (mAb) that binds to C5 (28). The clinical findings of severe TMA have also been observed in several participants who developed complement activation following systemic dosing of AAV after administration of Zolgensma for the treatment of SMA (30, 35). The finding of acquired hemophagocytic lymphohistiocytosis following Zolgensma therapy further demonstrates the broad impact of systemic AAV on immune activation (36, 37).

Multiple strategies to mitigate the immunologic response to AAV have been evaluated. These strategies include the administration of (a) high-dose glucocorticoids; (b) rituximab, an anti-CD20 mAb that depletes many B cell populations, resulting in impaired antibody production over time; (c) sirolimus, an mTOR inhibitor that assists in the inhibition of T and B cell activation; (d) plasmapheresis; and (e) cleavage of all circulating IgG antibodies (11, 34, 38). Given the complexity of the processes involved in the safety of systemic gene therapy, there is an urgent need to better understand and manage the mechanisms of complement activation following recombinant AAV administration.

In this study, we present a detailed time course and evaluation of complement activation in study participants receiving a single i.v. infusion of AAV9-mediated gene therapy. In addition, we characterize the complement profile in participants who received a systemic dose of AAV9 in conjunction with a targeted immune modulation regimen (IMR). When comparing these findings in Group 1 versus Group 2, we confirm our hypothesis that transient B cell and T cell immunomodulation (Group 2) prevents the most significant innate and adaptive immune responses following systemic high-dose AAV administration.

## Results

The comparative analysis of 2 groups using a distinct IMR was used to evaluate the immunological profile after administration of a therapeutic i.v. dose of AAV9 in 38 individuals (24 males and 14 females, age range 1 week to 11.7 years). The 2 groups consist of those who received only conventional corticosteroid dosing (Group 1, *n* = 23), which includes 16 participants (Group 1A) with SMA treated with Zolgensma and 7 participants (Group 1B) with Duchenne muscular dystrophy (DMD) receiving the investigational product from NCT03368742. Group 2 (*n* = 15) received rituximab plus sirolimus as the IMR in addition to corticosteroids to prevent the formation of anti-capsid antibodies and included 2 participants (Group 2A) with SMA treated with Zolgensma, 11 participants (Group 2B) with GM1 gangliosidosis (GM1) receiving the investigational product from NCT03952637 (NIH), and 2 participants (Group 2C) with Danon disease receiving the investigational product RP-A501 (NCT03882437). Table 1 describes demographics and treatment received in each group.

**Figure 1:**
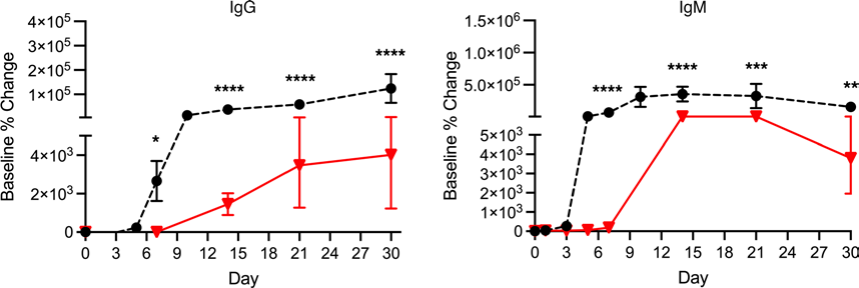
IgG and IgM antibodies against AAV9. Group 1 (dashed black lines and solid circles) received an i.v. dose of AAV9 without adjuvant immune modulation regimen (IMR). Group 2 (solid red lines and triangles) received IMR with rituximab and sirolimus before an intravenous dose of AAV9. IMR as an adjunctive therapy to AAV used in Group 2 prevented the formation of IgG and attenuated the formation of IgM. Data shown as mean ± SEM of Group 1 and Group 2 IgG and IgM baseline percentage change. *P* > 0.05 (nonsignificant), *P ≤ 0.05, **P ≤ 0.01, ***P ≤ 0.001, ****P ≤ 0.0001. Note: The split *y* axis is used to show the small response in Group2 compared with the 100-fold-higher antibody responses in Group 1.

**Figure 2:**
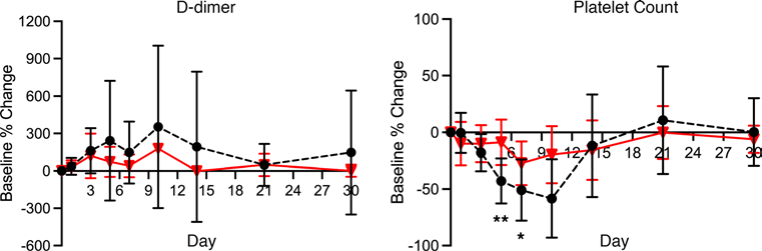
Hematology. Figure shows the percentage change from baseline for platelet count and D-dimer for Group 1 (dashed black lines and solid circles) and Group 2 (solid red lines and triangles). The data suggest that IMR as an adjunctive therapy to AAV used in Group 2 limits the depletion of platelets and the increase in D-dimer after AAV infusion. Data shown as mean ± SEM of Group 1 and Group 2 baseline percentage change for hematology. *P* > 0.05 (nonsignificant), *P ≤ 0.05, **P ≤ 0.01.

**Figure 3:**
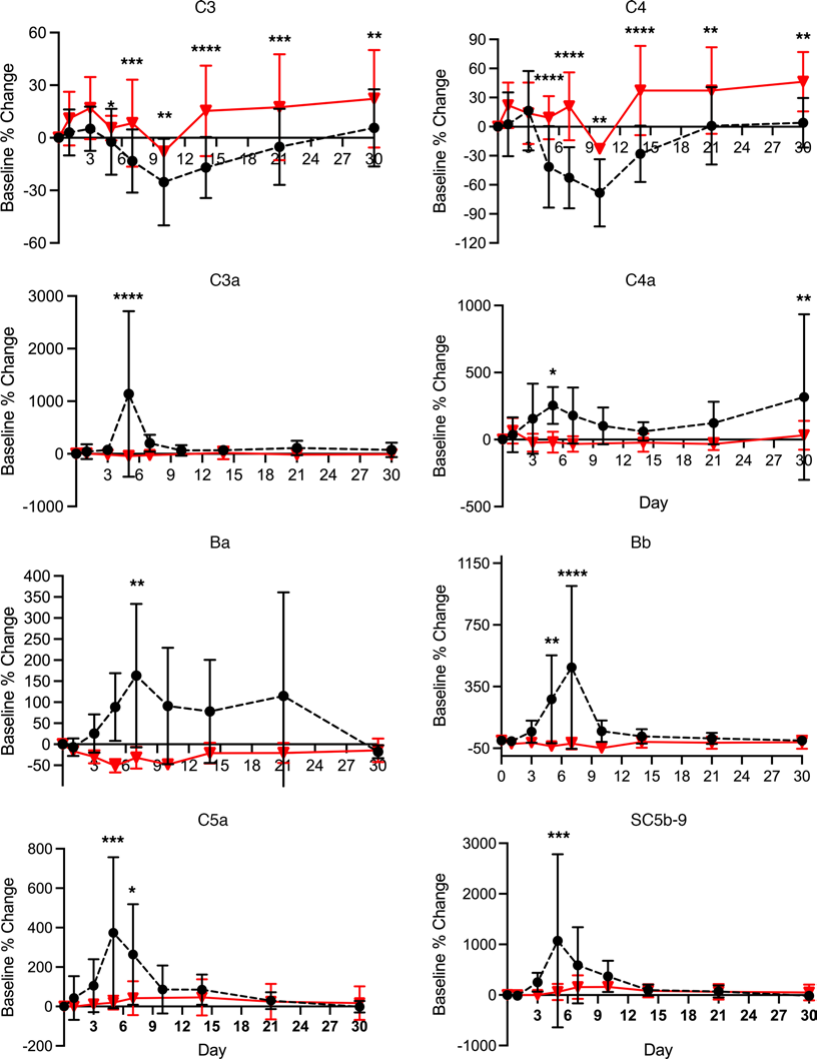
Complement system markers. Figure shows C3, C4, C3a, C4a, Ba, Bb, C5a, and SC5b-9 for Group 1 (dashed black lines and solid circles) and Group 2 (solid red lines and triangles). Participants in Group 1 presented with activation of the complement system, as demonstrated by a reduction in C3 and C4, as well as increased C3a, C4, C5a, Ba, Bb, and SC5b-9. The activation of Ba and Bb indicates that both classical and alternative pathway are involved. On the other hand, Group 2 did not show an activation of the complement system, suggesting that IMR as an adjunctive therapy to AAV prevents the activation of the complement system. Data shown as mean ± SEM of Group 1 and Group 2 baseline percentage change for complement. *P* > 0.05 (nonsignificant), *P ≤ 0.05, **P ≤ 0.01, ***P ≤ 0.001, ****P ≤ 0.0001.

**Figure 4:**
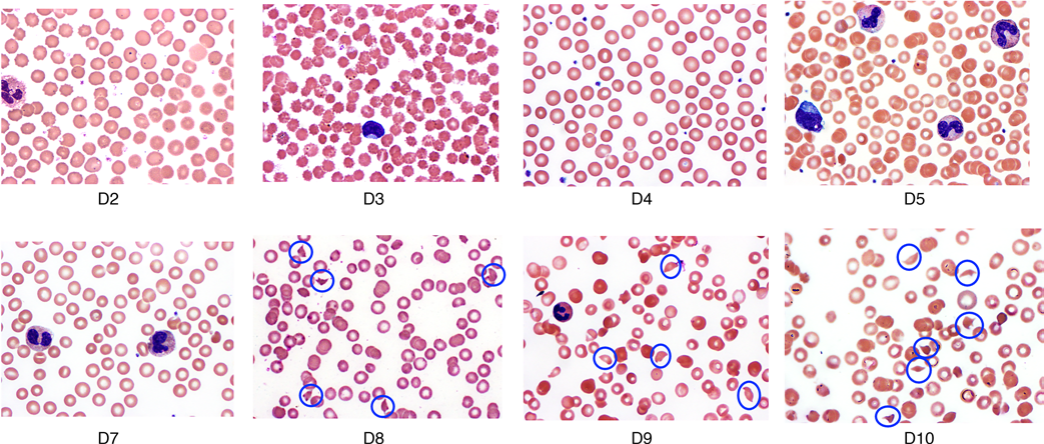
Peripheral blood smear. Sequence of peripheral blood smears after gene therapy administration in patient samples revealing increased schistocytes (blue circles), demonstrating evidence of endothelial damage, burr cells, polychromatic red blood cells, and thrombocytopenia, suggesting thrombotic microangiopathy. Blood smear of a patient with DMD (Group 1B) who received the investigational drug product in NCT03368742.

After AAV9 infusion, both IgM and IgG increased rapidly in Group 1 (prednisone or equivalent). Unlike any natural viral infection in which the first antigenic exposure is at the earliest stage of infection, the antigenic exposure to systemic AAV9 dosing is at a peak level within 1 hour of completing the infusion. Saturating levels of AAV capsids are found in every tissue compartment and certainly in all lymphoid tissue, possibly leading to a more rapid adaptive humoral response (KEC, unpublished observations). An increase in IgM level was first detected on day 5 after dosing and peaked on day 14. IgG was detectable by day 7 and continued to increase during the first 30 days after dosing. Group 2 participants received rituximab plus sirolimus IMR and there was no significant change in either IgM or IgG (Figure 1 and Table 2). The antibody increase in Group 1 coincides with a marked decline in platelet count and clinically significant thrombocytopenia in some participants. Thrombocytopenia has been observed in many individuals who receive systemic AAV, including serotypes other than AAV9. An additional component of the systemic immune response is the increase in fibrin degradation products represented by the finding of elevated D-dimer in plasma. Extensive platelet depletion or increased D-dimer was not observed in Group 2 (Figure 2 and Table 3). Importantly, Group 1 showed activation of the complement system, as demonstrated by reduction in C3 and C4, as well as increased C3a, C4a, C5a, Ba, Bb, and SC5b-9 (Figure 3). The additional activation of Ba and Bb indicates that both classical and alternative complement pathways were involved. In Group 2, there was no detectable activation of most of these complement components and there was limited C5b-9 activation observed, demonstrating that the activation of complement relies principally on the classical pathway and is antibody dependent (Figure 3, Table 4, and Table 5). Additionally, of the 38 participants, 22 (Group 1A, *n* = 13; Group 1B, *n* = 7; Group 2A, *n =* 2) had regular peripheral blood smears performed after gene therapy administration. Of those participants in Groups 1A and 1B, 6 presented with few to moderate schistocytes between days 7 and 21 and 2 were newborns. Schistocytes were not observed in blood smears for any participants in Group 2A (Figure 4).

## Discussion

The data presented here confirm that complement is activated in all participants receiving systemic AAV9 infusions without targeted immune management and results in severe inflammatory responses. TMA may be subclinical based on laboratory findings or in the setting of high viral load leading to serious adverse events. A previous in vitro study showed that complement activation is observed with all adenovirus serotypes tested, including serotypes 1, 3, 4, and 9 (39). In addition, Cichon et al. found that the presence of preexisting anti-AAV antibodies appeared to play an important role in triggering the complement system (39). Two key factors likely contribute to the complement response and differentiate therapeutic use of AAV from a natural infection. A unique aspect of systemic AAV gene therapy is the substantial total dosage administered within a brief infusion window. A systemic AAV exposure can effectively overpower the adaptive immune response, which normally mitigates the impact of the majority of viral infections in individuals with a functional immune system. Since vector dose is based on the vector genome titer of the drug product, there has been limited opportunity to evaluate the total protein exposure in systemic AAV dosing when there is not a specification for drug product protein concentration in the certificate of analysis. The micro BCA assay for total protein concentration is a reliable measure of total capsid antigen exposure. The total protein concentration of an AAV preparation is linked to the empty/full capsid ratio when the product purity is known and therefore allows for comparisons across various AAV products. A theoretical product of typical purity at a concentration of 2 × 10^13^ vg/mL has a capsid protein concentration of 200 to 300 μg/mL. Therefore, a systemic infusion of such an AAV9 drug product will deliver a minimum of 5 mg of capsid protein in a newborn and up to 100 mg of capsid protein in an adolescent. Such a high exposure is certainly unlike a natural viral infection, even in the setting of viral sepsis. The second distinguishing feature of therapeutic AAV use is that a systemic dose of AAV9 at doses greater than 1 × 10^13^ vg/kg will result in exposure to an enormous surface area of the capsid particles (exceeding 20 m^2^, nearly half of the pediatric lung surface area). The innate immune response just from the particle surface area alone is a potent stimulus for the adaptive immune response.

We have also demonstrated that the AAV capsid may lead to activation of the alternative complement pathway. The complement antigens Ba and Bb are markers of alternative pathway complement activation and were identified in Group 1 after infusion and remained elevated through day 7, suggesting direct interaction of C3 and AAV capsid proteins. The alternative pathway can serve as an amplification loop for classical pathway activation. While there is an antibody response to all AAV serotypes that can lead to IgM- and IgG-mediated activation of the classical pathway, there is a possibility that unique direct interactions of AAV9 with C3 and the potential for greater amplification via the alternative pathway are part of the pharmacodynamics of AAV9.

These findings confirm our hypothesis that transient B cell and T cell immunomodulation (Group 2) prevents the most significant innate and adaptive immune responses following systemic high-dose AAV administration. Some study limitations should be acknowledged. First, there is an uneven distribution of AAV product exposure between Groups 1 and 2 due to the doses utilized, and the participants’ age and size in the respective cohorts. Second, while all products are based on AAV9, the manufacturing processes differ between the various sponsors, resulting in potential differences in product quality including purity profiles and CpG content.

Other AAV serotypes can also result in complement activation. For example, complement activation was observed in adult participants with Fabry disease receiving a single i.v. administration of 4D-310 in combination with prophylactic oral corticosteroids (ClinicalTrials.gov NCT04519749 and NCT05629559). The capsid component of 4D-310 is 4D-C102, which is an AAV2 capsid variant developed through a Therapeutic Vector Evolution discovery platform; 4D-C012 was generated by insertion of a unique 10–amino acid peptide that is repeated on the capsid surface. A codon-optimized version of the full-length human *GLA* gene has been developed by 4D Molecular Therapeutics for the treatment of Fabry disease. Tests for complement activation markers performed at various time points demonstrated that 5 study participants developed either aHUS or TMA after receiving 4D-310 in combination with prophylactic corticosteroids. Classical pathway complement activation was confirmed by an increase in SC5b-9 levels. The alternative pathway was also activated confirmed by an increase in Bb starting approximately 7 days after 4D-310 dosing. Anti-capsid IgM was significantly elevated within the first 5 days in these participants. Of note, the single participant who had the most severe aHUS was found to have had alternative complement pathway activation at baseline, a finding that may predict increased sensitivity to AAV-mediated induction of aHUS and/or TMA. In vitro complement activation assays demonstrated that the 4D-310 capsid (C102) does not directly activate complement in the absence of anti-capsid antibodies. These findings suggest that complement activation after 4D-310 dosing was mainly driven by IgM binding to C1q. This is consistent with the results of a comparative analysis of clinical trials of systemic administration of AAV9 described in this report.

### Immunosurveillance after AAV gene therapy.

**Figure 5:**
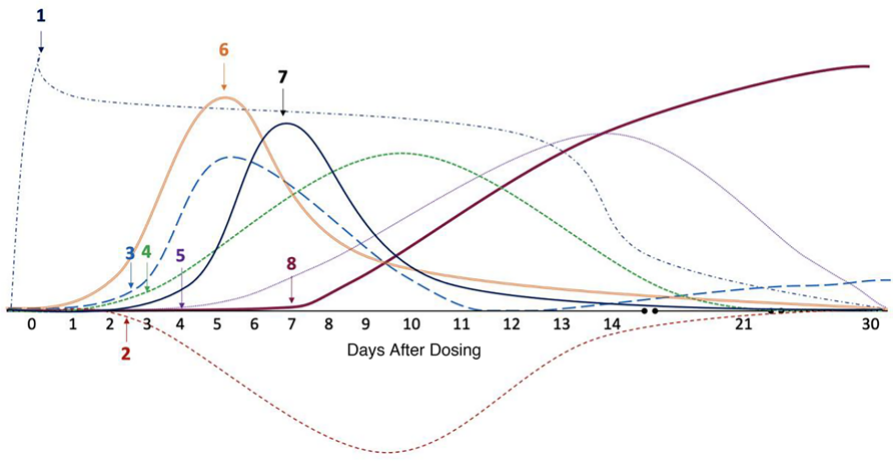
Immunological event timeline. Events: (1) AAV dosing and capsid biodistribution; (2) platelet, C3, and C4 depletion; (3) aspartate aminotransferase (AST) and alanine aminotransferase (ALT) elevation; (4) D-dimer elevation; (5) IgM elevation; (6) SC5b-9, C5a, C3a, and C4a elevation; (7) Bb, Ba, and factor I elevation; and (8) IgG elevation. The curve for each parameter was created using the average values obtained in the corticosteroid monotherapy group. The curves of each parameter were overlaid on the graph to show the peaks and trough over time, creating a timeline of events. The amplitude of each curve was adjusted to fit all the curves in 1 graph. The ratio of the peaks’ amplitude was maintained from the original data.

This report highlights the importance of frequent immunosurveillance during the first 30 days after AAV dosing, including comprehensive complement and hematology panels, D-dimer, and other indicators of endothelial activation. In addition, the total anti-AAV antibody levels should be measured before and after administration of AAV gene therapies since measuring only the titer of neutralizing antibodies provides very little information about the total amount of complement-activating antibodies, such as IgM (40). As a result of the detailed kinetic profile of the innate and adaptive immune responses, we have identified the key time points for optimal evaluation of the rate of change from baseline and early identification of potential serious adverse events; the interval between days 4 and 10 after AAV dosing is really the critical period, and values on day 7 alone may not enable even the most diligent clinician to conclude that the risk of TMA is low. We have modeled the immunological event timeline in (Figure 5). Tracking the clinical status and predictive laboratory indicators will enable investigators and clinicians using future commercial gene therapy products to monitor and anticipate clinical findings, which would allow for time to increase observation, either as an outpatient or in hospital. Importantly, we have identified an approach for pretreatment using rituximab and sirolimus that protects from an early IgM and IgG response that is the key trigger to safety events in the first week following systemic gene therapy. Early recognition of the immunological events described will allow for the opportunity to implement other countermeasures, especially for the prevention and management of adverse cardiovascular events. Systemic gene therapy is one of the few medical therapies devised to date, akin to solid organ transplant, that cannot be reversed (41), therefore access to detailed safety data will help establish best practices and improve the safety of this transformative therapy.

## Methods

All samples received from the NIH for complement and cytokine analysis were deidentified and under a Confidentiality Agreement for Coded Biologic Specimens and Data. Investigations before and after gene therapy were performed as follows: All participants had baseline labs drawn either on the day before to or on the day of infusion prior to any gene therapy administration (day 0). All participants received a single i.v. infusion of an AAV9 gene therapy product. Following the infusion, labs were drawn on days 3, 5, 7, 14, 21, and 30 after gene therapy administration as the minimal data set. Additional visits were scheduled if needed based on clinical condition or laboratory abnormalities. All labs through week 1 were drawn through a peripherally inserted central catheter that had been placed prior to gene therapy infusion.

### Adjunctive immunomodulation to prevent antibodies against AAV.

Group 2 received prophylactic adjunctive immunomodulation therapy before AAV dosing to prevent antibody development against AAV. Participants received a total of 1500 mg/m^2^ rituximab i.v. (Rituxan), divided in 2 to 4 doses received up to day –1 prior to AAV9 infusion. Prior to each rituximab dose, the participants received premedication with oral doses of acetaminophen (15 mg/kg), diphenhydramine (1 mg/kg), and methylprednisolone (1 mg/kg). The participants also received daily oral sirolimus (Rapamune, 0.5–1 mg/m^2^/d, adjusted to maintain a trough level of 3–7 ng/mL) starting the day before AAV9 infusion and continuing until day 180 after gene therapy administration.

### Corticosteroid treatment and additional immunosuppressive drugs.

Participants in Group 1A (SMA) and 2A (SMA) received prophylactic oral prednisone (1 mg/kg/d) 1 day prior to AAV gene therapy administration and continued for 4 weeks after infusion as per the drug insert with consecutive tapering per clinician’s discretion over 4 to 8 weeks. Six of the 7 subjects in Group 1B (DMD) received prophylactic oral prednisone (1 mg/kg/d) 1 week prior to AAV administration and continued for 12 weeks with consecutive tapering. The remaining participant in Group 1B received 2 mg/kg/d oral prednisone 1 day prior to and continued at a lower dose for 12 weeks after AAV administration.

In Group 1B (DMD), additional immunosuppressive drugs were used alongside glucocorticoids, including the mAb eculizumab that targets C5 (*n* = 6), the C1 esterase inhibitor Berinert (*n* = 3), and anakinra, an anti–IL-1 receptor antagonist to further block the inflammatory response against AAV (*n =* 1).

In Group 2B, 3 of the 11 participants did not receive corticosteroids and the remaining 8 received 0.5 mg/kg oral prednisone for 3 days following gene therapy administration. Along with oral glucocorticoids, Groups 1B and 2 received 1 mg/kg i.v. methylprednisolone at least 2 hours prior to AAV dosing. A summary of corticosteroid dosing and AAV9 dose is included in Table 1.

### Laboratory assays.

Complete blood count, kidney and liver function tests, coagulation panel, troponin I levels, C3, C4, and SC5b-9 levels were measured at the time points listed above. Any residual serum or plasma that was left over after all clinical labs had been completed was collected for further complement analysis and biobanking.

### Antibody assay.

Total anti-AAV9 IgG and IgM levels in serum were evaluated by enzyme-linked immunosorbent assays (ELISAs). Serum samples from the participants were assayed for circulating antibodies against AAV9 capsid. Briefly, 96-well plates were coated with 1.2 × 10^9^ AAV9 particles per well in sodium bicarbonate buffer, pH 8.4, overnight at 4°C. Subsequently, the plates were washed with a solution containing PBS and 0.05% Tween 20 (PBST) and then blocked with 10% fetal bovine serum (FBS; Cellgro) for 2 hours at 37°C. After being washed with PBST, the samples were serially diluted from 1:10 to 1:10,240 with a known positive human standard and allowed to bind overnight at 4°C. The plates were washed again, followed by addition of a secondary antibody (goat anti–human IgG or IgM conjugated with horseradish peroxidase [HRP]; Invitrogen) at a dilution of 1:20,000 for 2 hours at 37°C. Finally, the plates were washed and incubated with 3,3′,5,5′-tetramethylbenzidine (TMB) peroxidase substrate (Seracare Life Sciences) in the dark. Reactions were stopped with 0.1 M phosphoric acid. The reaction product was measured by spectrophotometric absorbance at 450 nm using a Gen5 Microplate Reader and Imager Software (BioTek Instruments). Sample titers were calculated using the mean absorbance of up to 3 dilutions that were within the linear region of a 4-parameter logistic standard curve generated by a known positive human standard.

### Multiplex complement assay.

Individual complement proteins Ba, Bb, C3a, C4a, C5a, SC5b-9, factor H, and factor I were analyzed simultaneously in duplicate in plasma or serum samples using the MicroVue Complement Multiplex – Standard 8-plex (Quidel, A900) according to the manufacturer’s instructions. Briefly, human serum (1:100 dilution), plasma (1:100 dilution), high and low controls, or assay calibrators were added to microplate wells arrayed with analyte-specific antibodies that captured Ba, Bb, C3a, C4a, C5a, and SC5b-9, thereby immobilizing Ba, Bb, C3a, C4a, C5a, and SC5b-9 on their respective locations within the array. The factor H and factor I competitive immunoassay reactions occurred simultaneously with the Ba, Bb, C3a, C4a, C5a, and SC5b-9 sandwich immunoassays. The factor H and factor I assays used capture antibodies specific for their respective targets. Human serum (1:100 dilution), plasma samples (1:100 dilution), high and low controls, or assay calibrators were added to microplate wells arrayed with immobilized analyte-specific antibodies that capture factor H and factor I. During the sample incubation, factor H present in a sample competed with a fixed amount of biotin-labeled factor H for sites on the immobilized antibody. In the same step, the factor I present in sample complexed with binding sites found on its respective immobilized antibody. In the subsequent detection step, biotin-labeled factor I was added and allowed to fill all available factor I antibody-binding sites. Following wash steps to remove excess biotin-labeled factor I and factor H and unbound protein, HRP was added to the microplate. After an additional wash, the amount of HRP remaining on each location of the array was inversely proportional to the amount of factor H and factor I initially present in a sample. The amount of conjugated enzyme on each location of the array was measured with the addition of a chemiluminescent substrate, read on a Q-View Imager LS (Quansys) and analyzed using Q-View software v3.12.

### Statistics.

Data were summarized using descriptive statistics. Percentage change from baseline values for parameters were analyzed using mixed-effects models for repeated measures with treatment group, visit, and treatment-group-by-visit interaction as fixed effects; and baseline values were included as a covariate for analyses on percentage change from baseline. A compound symmetry within-subject covariance matrix was assumed. SAS v9.4 software (SAS Insititute) was used for the modeling. A *P* value of 0.05 or less was considered significant.

### Study approval.

This study was approved by the University of Florida Institutional Review Board.

## Author contributions

SMS, MC, KEC, JAB, RWH, LRS, CLA, MEE, JDS, and BJB designed the study. SMS, JAB, PD, CJT, and CLA collected patient data. SMS, MC, KEC, MBW, JAB, PD, CJT, RWH, MEE, LRS, CLA, JDS, FT, DHK, and BJB analyzed and interpreted the data. MC wrote the first draft of the manuscript. All authors agreed on the content of the manuscript, reviewed drafts, and approved the final version. All authors had full responsibility for the decision to submit for publication. SMS and MC are co–first authors. SMB is listed first because she initiated the detailed data collection for this study and presented the initial findings at a national meeting and collected the data. MC contributed to the overall project supervision and concept as well as the majority of the data presentation.

## Supplementary Material

ICMJE disclosure forms

Supporting data values

## Figures and Tables

**Table 4:**
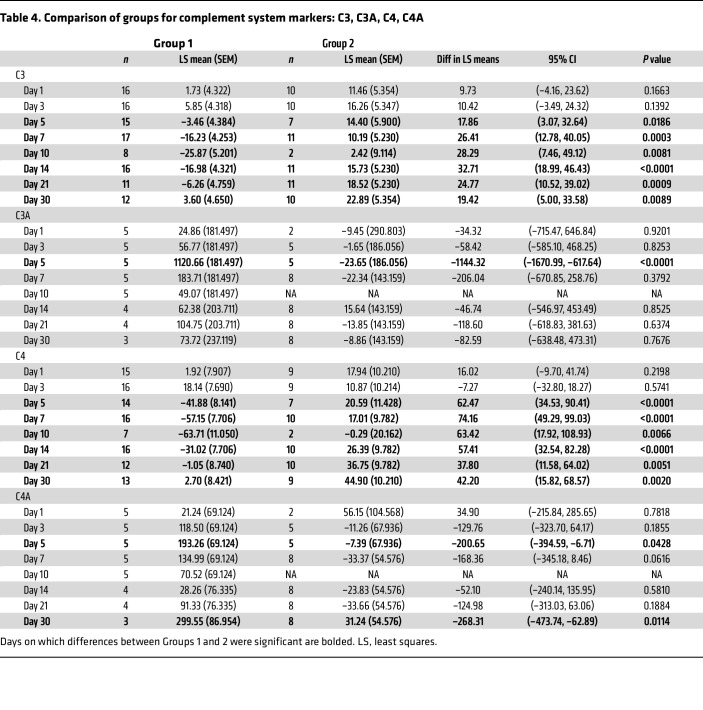
Comparison of groups for complement system markers: C3, C3A, C4, C4A

**Table 3:**
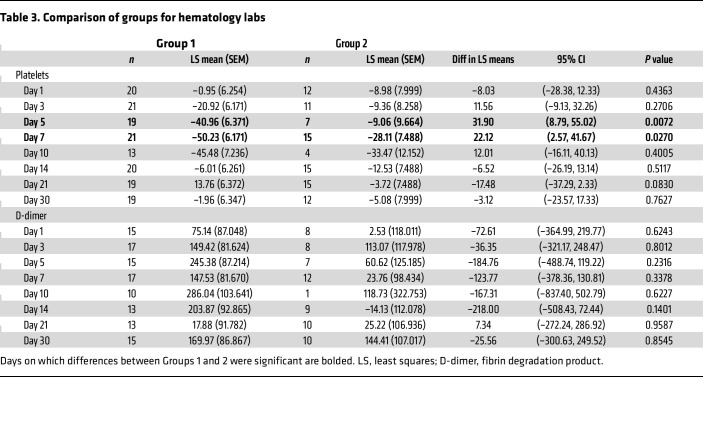
Comparison of groups for hematology labs

**Table 2:**
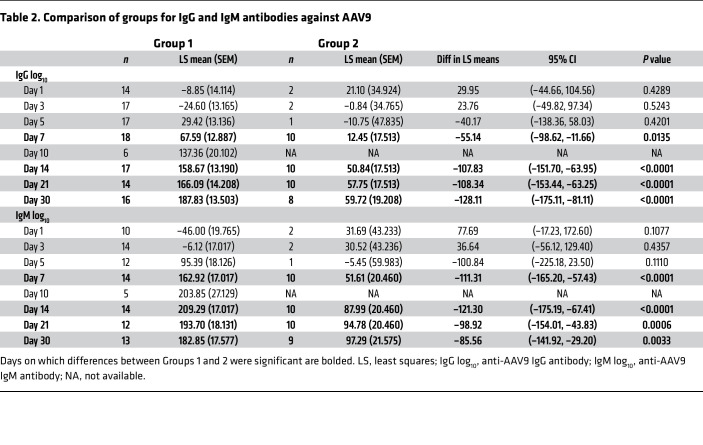
Comparison of groups for IgG and IgM antibodies against AAV9

**Table 1:**
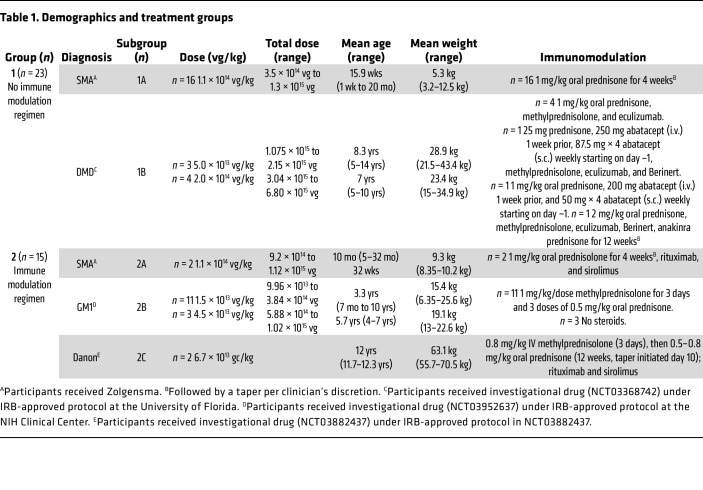
Demographics and treatment groups

**Table 5:**
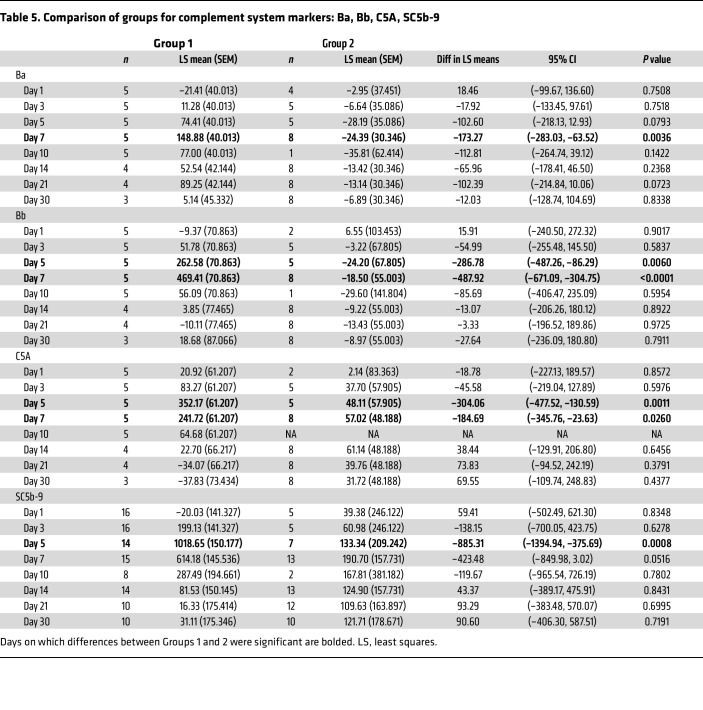
Comparison of groups for complement system markers: Ba, Bb, C5A, SC5b-9

## Version 1 11/21/2023

In-Press Preview

## Version 2 01/02/2024

Electronic publication
